# Formative Evaluation and Adaptation of a Hypertension Extension for Community Health Outcomes Program for Healthcare Workers within the Federal Capital Territory, Nigeria

**DOI:** 10.5334/gh.1277

**Published:** 2023-11-27

**Authors:** Abigail S. Baldridge, Nadia Goldstar, Grace C. Bellinger, Abigail T. DeNoma, Ikechukwu A. Orji, Gabriel L. Shedul, Rosemary C. B. Okoli, Nanna R. Ripiye, Adaora Odukwe, Olabisi Dabiri, L. Nneka Mobisson, Dike B. Ojji, Mark D. Huffman, Namratha R. Kandula, Lisa R. Hirschhorn

**Affiliations:** 1 Northwestern University, Feinberg School of Medicine, Chicago, Illinois, USA; 2 Havey Institute for Global Health, Northwestern University, Feinberg School of Medicine, Chicago, Illinois, USA; 3 Johns Hopkins University School of Medicine, Baltimore, Maryland, USA; 4 University of Abuja Teaching Hospital, Abuja, Nigeria; 5 University of Nigeria, Nsukka, Nigeria; 6 mDoc Healthcare, Lagos, Nigeria; 7 University of Abuja, Abuja, Nigeria; 8 The George Institute for Global Health, Sydney, Australia; 9 Washington University St. Louis, St. Louis, Missouri, USA

**Keywords:** hypertension, implementation, qualitative, primary care, education

## Abstract

**Background:**

The Extension for Community Health Outcomes (ECHO) model has been used extensively to link care providers in rural communities with experts with the aim of improving local patient care.

**Objective:**

The aim of this qualitative research study was to assess the feasibility, acceptability, perceived needs, and contextual factors to guide implementation of a hypertension focused ECHO program for Community Health Extension Workers (CHEWs) in the Federal Capital Territory, Nigeria.

**Methods:**

From September 2020 to December 2020, key informant interviews were performed with seven global organizations (hubs) providing ECHO training focused on cardiovascular disease or nephrology to identify contextual factors and implementation strategies used by each hub. In February 2022, seven focus group discussions were performed with 42 frontline healthcare workers in the Federal Capital Territory to inform local adaptation of a hypertension ECHO program. Directed content analysis identified major themes which were mapped to the Consolidated Framework for Implementation Research. Qualitative analyses were performed using Dedoose, and results were synthesized using the Implementation Research Logic Model.

**Results:**

We found both barriers and facilitators across the Consolidated Framework for Implementation Research domains that mapped to a number of constructs in each one. The results of these analyses confirmed that the core components of the ECHO model are a feasible and appropriate intervention for hypertension education of healthcare workers. However, implementing the ECHO program within the Federal Capital Territory may require strategies such as utilizing communications resources effectively, developing incentives to motivate initial participation, and providing rewards or recognition for ongoing engagement.

**Conclusions:**

These results provide valuable formative insights to guide implementation of our proposed hypertension ECHO program for CHEWs in the Federal Capital Territory, Nigeria. This information was used for key decisions around: 1) scope and content of training, 2) format and frequency, 3) selection of implementation strategies, and 4) building a community of practice.

## Introduction

Hypertension is a major health challenge in Nigeria, with an estimated 25–40% prevalence among adults based on blood pressure ≥140/90 mmHg or using blood pressure lowering drugs [1,2]. Management of hypertension is complicated by limited availability of healthcare workers trained and available to diagnose and treat the condition at primary care levels. To address this issue, the Hypertension Treatment in Nigeria Program (NCT04158154) is currently evaluating the implementation and effectiveness of hypertension service integration within 60 primary healthcare centers in the Federal Capital Territory, Nigeria, using an interrupted time series, type II hybrid design [3,4,5]. At the participating primary healthcare centers, hypertension patient care is delivered by trained, salaried, and supervised Community Health Extension Workers (CHEWs), supported by supervising community health officers and regional primary care physicians.

Before the Hypertension Treatment in Nigeria Program commenced, in-person training was provided at the University of Abuja Teaching Hospital (January 2020 to March 2020). These sessions aimed to educate two to four CHEWs from each participating primary healthcare center. The training included a two-hour didactic review of the program and an in-depth six- to eight-hour introduction to cardiovascular disease, blood pressure measurement, and hypertension treatment. Training materials were sourced from the Centers for Disease Control and Prevention Hypertension Management Training Curriculum and the World Health Organization [6]. Evaluation of the training revealed a relatively high knowledge of basic hypertension treatment and management, but identified gaps in knowledge relating to complex hypertension (**Supplemental Table 1**). Subsequent in-person and hands-on training sessions and quarterly site supervision visits by Hypertension Treatment in Nigeria Program study team members aimed to address these knowledge gaps.

The current model of in-person training is unsustainable for ongoing education, knowledge reinforcement, and training new CHEWs and other healthcare workers (HCWs). Contemporary and sustainable professional education among frontline HCWs to provide high-quality and robust hypertension care may be achieved through implementation of a telementoring program, such as the Extension for Community Health Outcomes (ECHO) model [7,8,9]. The ECHO model utilizes technology to leverage scarce resources, shares best practices to reduce disparities, employs case-based learning to master complex scenarios, and incorporates evaluation and monitoring in clinical settings. In the United States, ECHO hubs have successfully been used for hypertension education among primary care providers in urban settings and federally qualified healthcare centers, resulting in knowledge and self-efficacy improvements of 15–35% [8,9,10]. The ECHO model has been extended to community health workers; however, hypertension has not been formally included in this expansion to date [11]. Similarly, while the ECHO model has been used within Africa for other disease areas, its application among CHEWs has been rare [12,13].

Concurrent with the ongoing Hypertension Treatment in Nigeria Program, we proposed to adapt, implement, and evaluate a hypertension ECHO program for CHEWs within enrolling primary healthcare centers in the Federal Capital Territory, Nigeria. We conducted this qualitative formative study to assess the feasibility, acceptability, perceived needs, and contextual factors for implementation, and to guide adaptation and selection of implementation strategies for the hypertension ECHO program. This innovative application of the ECHO program has the potential to improve primary care-based management of hypertension Nigeria by equipping HCWs with necessary knowledge and skills through telementoring.

## Methods

### ADAPT-ITT framework

**Figure 1:**
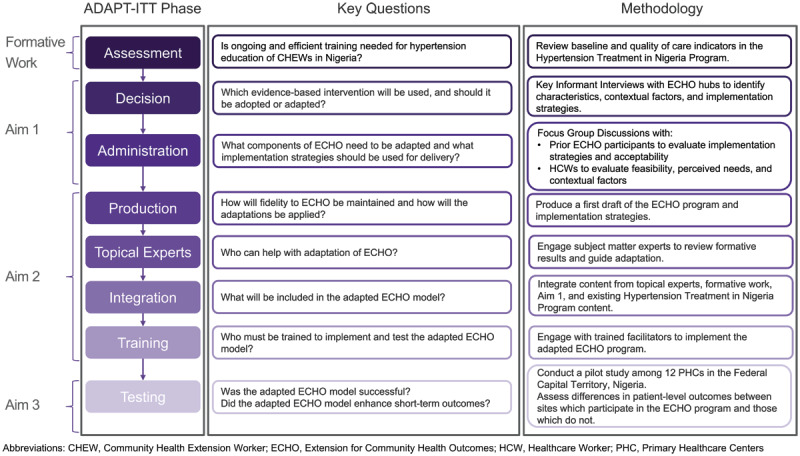
The ADAPT-ITT Framework for Hypertension Education of Community Health Extension Workers. *Legend:* The ADAPT-ITT framework provides a systematic approach for decision making and adaption of an evidence-based intervention. The Extension for Community Health Outcomes (ECHO) model was selected for hypertension education of Community Health Extension Workers (CHEWs) working in public primary healthcare centers in the Federal Capital Territory, Nigeria. Adaptation of the ECHO program focused on content, delivery, and implementation strategies followed the ADAPT-ITT framework.

The ECHO model was prospectively selected as an evidence-based intervention for delivery of hypertension training to CHEWs in the Federal Capital Territory, Nigeria. Adaptation, implementation, and evaluation of the hypertension ECHO program follows the ADAPT-ITT framework, which guides systematic adaptation of evidence based interventions (Figure 1) [14]. In this qualitative study, we report on the decision and administration phases (Table 1). In the decision phase, key informant interviews (KIIs) were performed first with ECHO hubs to understand their characteristics and operations and guide our implementation. Focus group discussions (FGDs) were subsequently conducted with local prior ECHO participants and frontline HCWs to assess readiness, identify barriers and facilities, and inform selection of implementation strategies.

**Table 1:** Qualitative Research Activities to Support Adaptation and Implementation of a Hypertension Extension for Community Health Outcomes Program for Community Health Extension Workers in the Federal Capital Territory, Nigeria.


COMPONENT	IDENTIFIER	PARTICIPANTS (ROLE)	FORUM	DATE

**Key Informant Interviews with ECHO Hub Administrators**				

	KII1	1 (Administrative)	Zoom	August 2020

	KII2	1 (Administrative) 1 (Medical Director)	Zoom	August 2020

	KII3	1 (Cardiologist)	Zoom	September 2020

	KII4	1 (Administrative)	Zoom	October 2020

	KII5	1 (Administrative)	Zoom	November 2020

	KII6	2 (Administrative)	Zoom	November 2020

	KII7	1 (Administrative)	Zoom	December 2020

**Focus Group Discussion with Healthcare Workers**				

	FGD1	5 (CHEW)	UATH	February 2022

	FGD2	3 (CHEW) 2 (CHO)	UATH	February 2022

	FGD3	7 (CHEW)	UATH	February 2022

	FGD4	3 (CHEW) 2 (Record Officer) 1 (Nurse)	UATH	February 2022

	FGD5	7 (CHEW) 1 (CHO)	UATH	February 2022

	FGD6	5 (CHEW)	UATH	February 2022

	FGD7	3 (Medical Doctor)3 (Nurse)	UATH	February 2022


*Abbreviations:* CHEW, Community Health Extension Worker; CHO, Community Health Officer; UATH, University of Abuja Teaching Hospital; KII, Key Informant Interview; FDG, Focus Group Discussion.

### Study setting, design, and sampling

#### Key informant interviews with ECHO hubs

Project ECHO hosts a global public database of all active ECHO hubs and the domains in which each hub offers trainings [15]. We searched the database in June 2020 to identify ECHO hubs that self-reported offering virtual cardiovascular and/or nephrology trainings. Representatives of each hub were invited to complete an electronic survey administered through REDCap, which consisted of questions about the characteristics of the hub including geographic location, years of functioning, number of staff and/or volunteers, number of trainings offered, and types of cardiovascular and nephrology trainings available (**Appendix A**) [16,17]. Hubs that specifically self-reported offering hypertension training in the electronic survey and were currently functioning (defined as active provision of or plans for training events) were contacted to schedule a key informant interview. ECHO hubs were contacted by email, website, social media, or phone at least three times at both the survey and interview stage before being considered nonresponsive. Interviews were conducted over Zoom by two female academic researchers (ASB, ATD) with master’s degrees in research related fields and familiarity with qualitative research methods. KIIs followed a semi-structured interview guide, including an introduction for the participant about the goals and reasons for conducting the interview. The KII interview guide focused on the implementation and function of the hub, barriers and facilitators to operations, target audiences for educational offerings, stakeholders, curricula, pedagogy, and methods for program evaluation (**Appendix B**). Neither interviewer knew any of the interview participants.

#### Focus group discussions with local prior ECHO participants and frontline HCWs

An ECHO program focused on non-communicable diseases was offered in 2021 by mDoc Healthcare – an ECHO hub based in Lagos, Nigeria – and advertised on social media with an open invitation for participation. Members of the Hypertension Treatment in Nigeria Program team and clinicians at the University of Abuja Teaching Hospital who were known to have joined one or more ECHO sessions were invited to participate through convenience sampling. A single FGD was conducted with the aim of understanding necessary adaptations to pedagogy and delivery of the proposed hypertension ECHO program.

In central Nigeria, the Federal Capital Territory is divided into six local government area councils, across which there are over 240 primary healthcare centers. Sixty of these primary healthcare centers were previously selected through a multi-stage, stratified random sample for the Hypertension Treatment in Nigeria Program [5]. Sites were ineligible to participate in the pilot hypertension ECHO program if they were selected for participation in the home blood pressure monitoring arm (n = 10 sites) of the Hypertension Treatment in Nigeria Program that was integrated into the overall study, or if they had a median of fewer than 25 recorded patient visits per month from March 2020 to September 2021 (**Supplemental Figure 1**). Among the 33 sites that were eligible to participate, stratified random sampling was used to select two primary healthcare centers from each local government area council. Six FGDs were conducted; one within each local government area council. All HCWs within the selected primary healthcare centers were invited to participate.

The University of Abuja Teaching Hospital research team recruited HCWs by phone. Team members contacted HCWs, explained the study aims and procedures, and assessed interest in participation. The HCWs who participated in the FGDs were all adults (age 18 years or older), able and willing to provide informed consent, and English-speaking. The experience and seniority of HCWs who agreed to participate were documented.

FGDs were conducted in English (the official language in Nigeria), by Hypertension Treatment in Nigeria Program members trained and experienced in qualitative research (IAO, GLS, NRR, RCBO). The interviewers consisted of both male and female academic researchers (primary care physicians: IAO, GLS, NRR; qualitative research specialists: RCBO) who knew the FGD participants through the Hypertension Treatment in Nigeria Program. The FGD guides included introductions for the participants about the goals and reasons for conducting the FGDs. All HCWs were able to speak comfortably in English, and no translation was needed. FGDs were centrally completed at University of Abuja Teaching Hospital, and participants were compensated with 2000 Naira as well as the cost of transportation. All participants provided written informed consent to participate and for audio recording. After providing informed consent, participants shared brief demographic information (e.g., primary health center, age, gender, years of experience, and position), which was documented on paper. We used the Consolidated Framework for Implementation Research (v1.0) to structure the FGDs, and prospectively developed questions based on selected domains (**Appendix C** and **Appendix D**) [18]. Each FGD comprised a 90-minute discussion to understand HCWs’ perceptions of existing hypertension trainings, and the needs, feasibility, and acceptability for additional hypertension training.

### Data management and analysis

KIIs and FGDs were audiotaped and transcribed verbatim by Zoom (KIIs) or QualTranscribe (FGDs). Transcripts were read several times by study team members and were not shared outside of the analytic team. A codebook for the KIIs was developed for deductive coding based on the interview guide, and KIIs were analyzed by a single reviewer (ASB). Codebooks for the FGDs were developed based on Consolidated Framework for Implementation Research constructs from the interview guides, and analysis followed a directed content approach. Each FGD was analyzed in duplicate (ASB, GCB, and NG), with discrepancies resolved through consensus coding. Dedoose software (v8; Socio-cultural Research Consultants) was used to assist with organizing and analyzing the qualitative data. Major themes across the KIIs and FGDs were organized under Consolidated Framework for Implementation Research constructs and identified as either barriers or facilitators of the prospective hypertension ECHO program with CHEWs. These were then mapped to strategies using mechanism mapping and documented with the implementation science tool, Implementation Research Logic Model [19].

Self-reported participant characteristics were summarized using SAS (SAS v9.4, Cary, NC). Characteristics are reported as median and interquartile range for continuous variables and frequency and percentages for categorical variables.

### Ethics and reporting

The reporting of this study adheres to the Consolidated Criteria for Reporting Qualitative Research guidelines [20]. The protocol for surveys and interviews with ECHO hubs was reviewed by the Northwestern University Institutional Review Board (STU00212906) and determined to be exempt as non-human subjects’ research. The protocol for FGDs with prior ECHO participants and CHEWs in the Federal Capital Territory, Nigeria was reviewed and approved by the Ethics Committee at the University of Abuja (UATH/HREC/PR/2021/011/015) and determined to be exempt by the Northwestern University Institutional Review Board (STU00216041).

## Results

### Participant characteristics

#### Key informant interviews with ECHO hubs

Twenty ECHO hubs were identified through the Project ECHO website, all of which self-identified a cardiovascular focus (**Supplemental Figure 2**). Two-thirds (n = 13, 65%) responded to the survey invitation. Those that did not respond were either non-functional (n = 3; 15%) or could not be reached after three attempts (n = 4, 20%). Most respondents were from hubs located in the United States (n = 8, 62%; Table 2). The median (interquartile range) length of time which the responding hubs had been functioning was 5 (3 to 8) years. The most commonly reported participants were nurses (n = 12; 92%); followed by nurse practitioners (n = 9, 69%); primary care, family, or geriatric physicians (n = 9; 69%); trainees (n = 8; 62%); and cardiologists (n = 7; 54%). Among the 13 hubs that responded to the survey, seven (54%) KIIs were conducted with hub representatives. Five hubs were excluded from the KIIs either because they did not offer hypertension training (n = 4) or because they were non-functional (n = 1), and one hub was lost to follow up after responding to the survey. Among the hubs interviewed, four (57%; n = 2 in the US, n = 1 in Australia, n = 1 in Ecuador) reported providing training to community health workers.

**Table 2:** Characteristics of Extension for Community Health Outcomes Hub Survey Respondents.


CHARACTERISTIC, NO. (%)	NO. RESPONSES	OVERALL (n = 13)	ELIGIBLE FOR INTERVIEW		NOT ELIGIBLE (n = 5)

			INTERVIEWED (n = 7)	NOT INTERVIEWED (n = 1)	

Location	13				

Australia		1 (8)	1 (14)	0 (0)	0 (0)

Ecuador		1 (8)	1 (14)	0 (0)	0 (0)

India		1 (8)	0 (0)	0 (0)	1 (20)

Ireland		1 (8)	0 (0)	0 (0)	1 (20)

United States		8 (62)	5 (71)	1 (100)	2 (40)

Uruguay		1 (8)	0 (0)	0 (0)	1 (20)

Primary Funding Source	13				

Government/Federal		2 (15)	2 (29)	0 (0)	0 (0)

International NGO		1 (8)	0 (0)	0 (0)	1 (20)

Local NGO		3 (23)	1 (14)	0 (0)	2 (40)

University		4 (31)	2 (29)	1 (100)	1 (20)

Other		2 (15)	2 (29)	0 (0)	0 (0)

None		1 (8)	0 (0)	0 (0)	1 (20)

Years functioned, median (IQR)	13	5 (3–8)	4 (3–9)	3 (NA)	6 (5–8)

Currently providing training	13	11 (85)	7 (100)	1 (100)	3 (60)

Full-time staff, median (IQR)	12	1 (1–3)	2 (1–3)	1 (NA)	1 (0–1)

Part-time staff, median (IQR)	12	2 (0–2)	2 (0–2)	1 (NA)	1 (0–4)

Types of training	13				

Hypertension		8 (62)	6 (86)	1 (100)	1 (20)

Heart Failure		7 (54)	3 (43)	1 (100)	3 (60)

Imaging		2 (15)	1 (14)	0 (0)	1 (20)

Coronary Artery Disease		2 (15)	1 (14)	0 (0)	1 (20)

Rhythm		3 (23)	2 (29)	0 (0)	1 (20)

Primary Prevention		6 (46)	4 (57)	1 (100)	1 (20)

Secondary Prevention		5 (38)	3 (43)	1 (100)	1 (20)

Nephrology		6 (46)	4 (57)	1 (100)	1 (20)

Training audience	13				

Cardiologists		7 (54)	3 (43)	1 (100)	3 (60)

Nephrologists		4 (31)	2 (29)	1 (100)	1 (20)

Primary, family, or geriatric physicians		9 (69)	6 (86)	1 (100)	2 (40)

Emergency medicine physicians		3 (23)	1 (14)	0 (0)	2 (40)

Nurse practitioners		9 (69)	5 (71)	1 (100)	3 (60)

Nurses		12 (92)	7 (100)	1 (100)	4 (80)

Technicians		2 (15)	1 (14)	0 (0)	1 (20)

Community Health Workers		5 (38)	4 (57)	1 (100)	0 (0)

Trainees		8 (62)	5 (71)	1 (100)	2 (40)

Other		3 (23)	3 (43)	0 (0)	0 (0)


*Abbreviations:* IQR, Inter-quartile Range; NA, Not Applicable; NGO, Non-Governmental Organization.

#### Focus group discussions with local prior ECHO participants and frontline HCWs

Characteristics of FGD participants are reported in **Supplemental Table 2**. Across seven FGDs, 42 HCWs participated, the majority of which (n = 30; 71%) were CHEWs and among which the median (range) age was 42 (24–58) years. Over half of the participants (n = 25; 60%) were female, and an identical proportion was employed full-time (n = 25; 60%). Occupation and education varied across groups with differences driven by intrinsic selection of medical doctors and nurses for the FGD with prior ECHO participants (i.e., FGD7). None of the prospective participants who were approached declined to participate.

### Key themes identified as determinants

**Figure 2:**
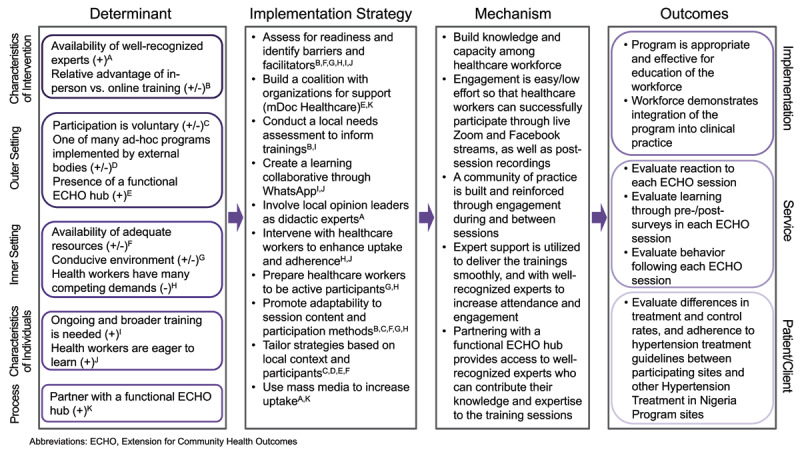
Implementation Research Logic Model for the Hypertension ECHO Program. *Legend:* Barriers (–) and facilitators (+) identified through key informant interviews and focus group discussions are mapped under the Consolidated Framework for Implementation Research as determinants. Each determinant is associated with a letter to indicate alignment with implementation strategies. Implementation strategies for the hypertension Extension for Community Health Outcomes (ECHO) program were selected to address barriers and align with facilitators. The proposed mechanism of action and planned outcomes evaluation are mapped to implementation, service, and patient/client level outcomes.

We found both barriers (–) and facilitators (+) across the Consolidated Framework for Implementation Research domains, all mapping to a number of constructs in each domain as outlined by the Implementation Research Logic Model (Figure 2). Quotes supporting each theme are included in Table 3.

**Table 3:** Themes and Quotes that Emerged from Formative Interviews about the Hypertension Extension for Community Health Outcomes Program among Frontline Healthcare Workers in the Federal Capital Territory, Nigeria.


CFIR DOMAINS AND THEMES	IDENTIFIER	QUOTE	BARRIER (–) OR FACILITATOR (+)

**Characteristics of Intervention**			

Availability of Well-Recognized Experts			

	KII1	“We [The Hub] just tried to build a multi-disciplinary hub. So we tried to have MDs on there with certain skill sets. For diabetes [a] diabetes educator, nutritionists, even social work or case manager or something of those sorts.”	+

	KII5	“We [The Hub] have Dr. [REDACTED] who is well known and his credentials are just phenomenal. In regards to high blood pressure and management.”	+

	KII5	“He is such a fabulous teacher, you can tell when he is in the zone when he does these echoes he is just right in that zone and the conversations that he can generate with the members is really pretty phenomenal.”	+

	KII4	“Our program is housed here in the Department of Pediatrics in [University] […] we [The Hub] do partner with over 60 facilitators who are within different departments”	+

	KII7	“We [The Hub] want to make sure we get really good didactic presenters that encourage good attendance and give the best education possible”	+

	KII4	“They [The Participants] are not just hearing from a specialist, but they’re also learning from the experiences of all of these other providers from different organizations.”	+

Relative Advantage of In-Person vs. Online Training			

	KII7	“And so ECHO really fits in nicely into that reaching out to practices across huge geographic distances and providing education in a way that general practice likes to receive education rather than a complete didactic session, giving them lots of space and comfort to contribute their knowledge.”	+

	FGD5	“Then once in a while, we [HCWs] can have the kind of training like this, let it not be only online. Because you know when you were asking some questions, you elaborated on it before we can answer it. So, sometimes, face-to-face discussions too will help.”	+/–

	FGD2	“Classroom training is always the best because there are possible interactions.”	–

	FGD2	“I will also prefer the classroom training because in the classroom, after the teaching, we [HCWs] can ask questions. Whatever we don’t understand, we can easily ask questions and get the answer back. I prefer that one.”	–

	FGD4	“I like that idea of staying alone to listen to it [training], rather than gathering.”	+

	FGD2	“I will suggest both classroom and Zoom because it is good to learn some things. You don’t remain in classroom all the time. It is good because even in that Zoom learning, you can ask questions. You can raise your hand and ask questions.”	+/–

**Outer setting**			

Participation is Voluntary			

	KII2	“People choose to show up […] right now most are monthly and kind of open, and you know, just kind of come when you can.”	+

	KII7	“We [The Hub] were seen to be a lot of effort. A lot of advertising for I think we had 21 people attend. We had over 40 register and then we got 21.”	–

	KII6	“It is hard to get them [The Participants] to participate, get their opinions and questions.”	–

	KII6	“Rural doctors are hard to convince, it’s sometimes hard to get cases to study, they don’t easily understand that these discussions will help to solve a life.”	–

One of Many Ad-Hoc Programs Implemented by External Bodies			

	FGD2	“They [Program Managers] should integrate hypertension program with diabetes program.”	–

	FGD7	“We [HCWs] are used to different trainings being organized online from time to time. So once we hear any program, as long as we are available, most often we key in because we know it’s still another avenue to improve our knowledge.”	+

Presence of a Functional ECHO Hub			

		A key finding during our conduct of KIIs was that numerous ECHO hubs listed by Project ECHO were non-functional at the time we contacted and surveyed them.	+

**Inner Setting**			

Availability of Adequate Resources			

	KII3	“We [The Hub] eventually overcame those [technological issues] and our technology now is much better.”	+

	KII2	“We [The Hub] just need a little bit more staff support […] it’s a lot nicer when you have more people obviously.”	+/–

	KII6	“We [The Hub] need a strong communications support.”	+/–

	FGD1	“This online training needs data. So if they [ECHO Program] can provide data to CHEWs, I think they [CHEWs] will be happy because the knowledge they will get is more than the financial aspect they will think of. But if they have data that they can connect, I think it will help.”	–

	FGD5	“If they [ECHO Program] can provide a tablet to use in facilitating information […] A tablet, mobile devices, it will help […] for the online, they will need a laptop or a tablet for that online meeting.”	+/–

	FGD5	“About the online training, it must not really be the laptop, it can be an android phone or something and you know, you can’t just use it like that without data, there must be data”	+/–

	FGD5	“What I know is that we [HCWs] need an android phone or a tablet or something if it will be individual. Like me now I don’t have an android phone and if anything is coming online I can’t afford it, so if it is individual, I won’t be able to participate, but if it is a group one, since you said our PHC has one, we will be able to do it together, so far as there is data for it.”	–

	FGD7	“The major challenge in participating in the online program is actually network fluctuations and power failure. Because some gadgets may not be able to go an hour or two online without the battery being drained. Whereas, when the network is not stable, especially during rainy season being the month of June-July, once it’s raining, definitely our [HCWs] ability to browse or assess online activity goes off.”	–

Conducive Environment			

	KII1	“The biggest barrier across all hubs, not even just endocrine is time, you know when’s the best time to have the sessions and then can the providers get off that time. It’s always the biggest barrier.”	–

	KII1	“You might have weeks that you [The Hub] might not get a case. And that’s typically just because the folks [The Participants] don’t have time to submit them or they cancel on you last second, or something like that because they can’t come to the session.”	–

	KII3	“Early on I think we [The Hub] noticed the attendance went down during the summer months because of vacations […] So we, at times would decide to take a break during the summer to allow summer vacations to occur.”	+/–

	KII6	“Sessions are delivered by Zoom synchronously. The sessions are not pre-recorded, and participants are supposed to participate live as that is better for overall participation and engagement.”	+

	FGD7	“The disadvantage, I will not say that I have any major disadvantage, but the timing of listening to that was an issue to me [Prior ECHO Participant]. People learn better early morning. Early morning when you wake up, you come by 8:00 a.m. If the presentation is delivered early and we come face-to-face early, you tend to listen more and you tend to understand more.”	–

	FGD7	“The advantages from the training that I [Prior ECHO Participant] had is that the timing is after the normal facility work, from 2:00 down, because we close by 2:00. From that 2:00 to 4:00, sometimes we’ll be free and we can now attend the meetings and listen.”	+

	FGD7	“And the place, like my [Prior ECHO Participant] PHC, where we gather to listen sometimes, maybe they might use the office. And then they will say, ‘We want to use this place for another thing.’ Then we’ll now begin to look for where to stay.”	–

	FGD7	“When it’s [The ECHO Program] coming, the person [Participant] should be left alone to join fully. Less work, less problem with less headache. You just concentrate and do it. We need time.”	+/–

	FGD7	“For those of us in the tertiary center, reminders, motivation, and encouraging us to join in the online program will go a long way to help us get interested and to participate fully in the program.”	+

Health Workers Have Many Competing Demands			

	KII4	“They [HCWs] were not interested in the topic, but because they had to commit some time to participate in the series and that time was the time away from their clinics and the patients.”	–

	KII3	“And they [HCWs] have a full day patient panel and one hour of a lecture was viewed by some as just an extension and one hour lost in the day to do my clinical work, so that was that was the tension within sort of our region.”	–

	FGD6	“And sometimes one of the challenges that it may not make it [ECHO Program] effective is when you [HCW] are in your facility and probably you are connected online receiving the training, you might have a patient that is coming to see you for one reason or the other.”	–

**Characteristics of Individuals**			

Ongoing and Broader Training is Needed			

	KII3	“I’ve [Hub Administrator] been practicing now thirty years and when I first started as a specialist there were specialists they dealt with the more challenging difficult cases.”	+

	KII6	“The curricula are developed in consultation with key stakeholders based on knowledge gaps within the community of practitioners.”	+

	FGD3	“We [HCWs] will need training in resuscitation of a patient that just suddenly falls down. That training is key […] If we’re trained on how to handle such cases, we will reduce sudden cases of death.”	+

	FGD3	“I [HCW] want to improve on pregnant women […] if you notice any pregnant woman who are hypertensive, we refer them to [REDACTED] and they may not go. Most of them don’t go. Immediately you refer them, they will just go and sit at home. So, I don’t know if there is anything you people will do, on that aspect, so that we can give them something, instead of referring them.”	+

	FGD5	“Most of us [HCWs] don’t even know the complication that may arise from hypertension. And most of them, you know some drugs are contraindicated, so they wouldn’t, up to now, some people will still be giving some drugs to pregnant women and not knowing it’s contraindicated to the pregnancy. So that is why they need this training.”	+

	FGD3	“Yes, 100 percent need. We [HCWs] need more training. If I should score it, 100 percent more training. The reason why [training should continue] is because we, as the CHEWs, our primary assignment, 70 percent is in the rural community. And most of these clients are in the community, so we are always with them, we are always at their doorstep, we are always the entry point to the healthcare system, so there is need.”	+

Health Workers are Eager to Learn			

	FGD3	“If there will be more programs or trainings, we’ll [HCWs] be grateful to acquire more knowledge.”	+

	FGD3	“Knowledge is always increased, and it will also help us [HCWs] handle our patients very well.”	+

	FGD1	“Also, much training based on Zoom. As we say, we’ll [HCWs] be given more training. This time they will make the training to be six months. They will make the training basically six months. I think it will be good so that it will boost the knowledge of us working in the PHCs.”	+

	FGD3	“There is that need [to continue training] because health is teamwork. There is that need that all other health professional to be also trained, so that we go with a very nice target to cover, in hypertensive treatment.”	+

	FGD7	“One, it’s an opportunity to connect with other facilities beside my primary facility. Secondly, we also cut across primary, secondary, and tertiary health institutions. Thirdly, it’s still another avenue to increase knowledge and impact one another. Fourthly, my patients will also benefit too, not only waiting until I have been seen, knowing fully well that other people will also do the same thing.”	+

**Process**			

Partner With a Functional ECHO Hub			

	KII6	“Connect with the Project ECHO team and use their resources and knowledge for building a hub.”	+


*Abbreviations:* CFIR, Consolidated Framework for Implementation Research; CHEW, Community Health Extension Worker; ECHO, Extension for Community Health Outcomes; FGD, Focus Group Discussion; HCW, Healthcare Worker; KII, Key Informant Interview; PHC, Primary Healthcare Centers.

#### Characteristics of intervention: availability of well-recognized experts (+)

Engagement with well-recognized experts brings credibility, expertise, and trust to the ECHO hub. Having well-recognized experts facilitate ECHO sessions makes the training sessions more valuable and ensures that participants receive reliable and high-quality information. Their involvement contributes to the overall success and effectiveness of the ECHO hub in addressing knowledge gaps and supporting the professional development of healthcare workers.

#### Characteristics of intervention: relative advantage of in-person vs. online training (+/–)

The ECHO hubs underscored the advantages of online training including increased accessibility, convenience, and the ability to reach a wider audience. Utilizing platforms (such as Zoom) for online training facilitates synchronous sessions where participants can actively engage, ask questions, and receive immediate responses, creating an interactive learning environment. Among FGD participants, there were mixed beliefs about the effectiveness, convenience, and feasibility of online training versus in-person training. Multiple participants suggested a hybrid approach. Most CHEWs agreed that having training in-person would be convenient and conducive to learning.

#### Outer setting: participation is voluntary (+/–)

Across the ECHO hubs who participated in KIIs, participation in the trainings was universally voluntary for health workers. Voluntary participation can empower health workers to choose relevant trainings and foster motivation, enthusiasm, and active engagement. On the other hand, some health workers may be hesitant or reluctant to participate voluntarily due to various reasons such as time constraints, workload, or skepticism about the benefits of the training. Challenges such as competing demands and potential resistance to voluntary participation should be addressed to ensure optimal involvement and maximize the benefits of the training programs. Efforts were made by the hubs to motivate participant involvement through rewards, incentives, and recognition.

#### Outer setting: one of many ad-hoc programs implemented by external bodies (+/–)

The presence of multiple ad-hoc programs can offer diverse training opportunities and access to different knowledge sources. However, challenges such as fragmentation, lack of coordination, sustainability issues, and competition among programs need to be considered to support HCWs’ training needs and effectively utilize resources. The existence of multiple ad-hoc programs can create a competitive environment among different initiatives, potentially leading to redundant efforts and inefficiencies in resource allocation.

#### Outer setting: presence of a functional ECHO hub (+)

The ECHO hub plays a crucial role in facilitating knowledge sharing, improving skills, and providing ongoing support for health workers. During our assessment of ECHO hubs, we found that four hubs (one each in Egypt, United States, Ireland, and India) had become non-functional over time, likely due to barriers such as funding, staffing, and competing initiatives – all of which were mentioned as challenges by the hubs we interviewed.

#### Inner setting: availability of adequate resources (+/–)

At the ECHO hub level, the availability of adequate resources is critical. Therefore, it is crucial to address any potential limitations or barriers to access to maintain uninterrupted operations. Beyond funding and staffing, having access to training curricula, case studies, and continuing education materials, enhances the learning experience and professional development of the participants. Several hubs mentioned challenges in obtaining specific case studies for the training sessions, which can hinder the depth of discussion and learning. FGD participants largely agreed that the effectiveness of the ECHO program would be contingent on the availability of adequate resources (e.g., space, internet at the training site, etc.) for them to participate.

#### Inner setting: conducive environment (+/–)

ECHO hubs strive to create a conducive environment for learning and knowledge sharing. This is achieved through interactive and participatory training sessions, open discussions, and opportunities for collaboration and networking. FGD participants identified that the effectiveness of the ECHO program would be contingent on lack of distraction, appropriate timing, alleviating competing clinical demands, as well as the availability of a quiet and private space. There were mixed opinions shared by HCWs on the timing of the training (i.e., during or after the workday).

#### Inner setting: health workers have many competing demands (–)

ECHO hubs acknowledged that health workers face multiple competing demands in their clinical work, which can make it challenging for them to actively participate in training programs. Hubs reported making efforts to accommodate HCW schedules and provide flexibility in training sessions. Healthcare workers noted that the ECHO program may not be effective if the participants are joining training sessions from the clinic during a time when patients are seeking care.

#### Characteristics of individuals: ongoing and broader training is needed (+)

The ECHO hubs recognize the need for ongoing and broader training to address the evolving healthcare landscape. The hubs aim to provide continuous learning opportunities, regularly revising training curricula based on identified knowledge gaps. A number of training gaps were identified by FGD participants, such as gestational hypertension, comorbidities, and emergency cases. HCWs also professed the need to have cross-trained staff which can seamlessly provide full coverage patient care in the case of individual absences from the healthcare center.

#### Characteristics of individuals: health workers are eager to learn (+)

ECHO hubs reported the positive impact of healthcare workers’ eagerness to learn. Their enthusiasm and motivation to acquire new knowledge and skills are valuable assets that drive continuous improvement in their practice. FGD participants similarly demonstrated eagerness to learn more about hypertension, and specifically noted that this would enable them to better care for their patients.

#### Process: partner with a functional ECHO hub (+)

Collaborating with a functional ECHO hub can provide a valuable network of expertise, support, and resources. Partnering with such hubs can enhance the effectiveness and sustainability of training initiatives. All ECHO hubs who were interviewed were partnered with one or more organizations for long-term support and had transient and focused partnerships with additional organizations, both global and local, for targeted trainings and support.

### Reflecting and evaluating

The results of these analyses confirmed that the core components of the ECHO model are a feasible and appropriate intervention for hypertension education of HCWs, and that the ECHO model itself (i.e., use of technology, sharing of best practices, application of case-based learning, evaluation and monitoring of outcomes) may be adopted without significant adaptation. Implementing the ECHO program within the Federal Capital Territory, Nigeria, for CHEWs may require adaptations to overcome resource constraints, such as developing incentives to motivate participation, providing rewards or recognition for active engagement, and utilizing existing resources effectively. It is critical to create a conducive environment for the ECHO program, including raising awareness among HCWs about the program’s benefits, addressing any resistance or skepticism, and fostering a culture of continuous learning and knowledge sharing.

### Selection of implementation strategies

These results were used to inform selection of implementation strategies for the hypertension ECHO program. We used mechanism mapping to identify strategies that would address barriers or leverage facilitators (Figure 2).

- *Build a coalition with organizations for support*: Partnering with an established ECHO hub that has experience and resources can provide valuable support and guidance during the implementation process. Collaborating with a functional ECHO hub can help leverage their expertise, established protocols, and access to well-recognized experts. We chose to collaborate with mDoc Healthcare for development and delivery of the hypertension ECHO program.
- *Conduct a local needs assessment to inform trainings*: Identify the specific knowledge gaps and training needs of healthcare providers in the specific setting. This assessment will help tailor the ECHO program to address the most pressing issues and increase its relevance and effectiveness.
- *Create a learning collaborative through WhatsApp*: Facilitate and encourage uptake and sustained engagement in the training sessions by empaneling HCWs in a WhatsApp group for promotion of the training sessions.
- *Involve local opinion leaders as didactic experts*: Engage local experts from healthcare providers, institutions, professional societies, and government agencies as experts to lead didactic sessions and facilitate case studies. Their involvement and input will contribute to engagement and reputation of the program.
- *Intervene with HCWs to enhance uptake and adherence*: Develop strategies to motivate and incentivize healthcare providers to actively participate in the ECHO program. This can include offering certificates of completion, providing continuing education credits, recognizing active participants through awards or recognition ceremonies, or providing credit for data usage.
- *Prepare HCWs to be active participants*: Engage selected sites to participate through case-based presentations to encourage active discussion and ownership in the ECHO sessions.
- *Promote adaptability to session content and participation methods*: Ensure that the ECHO sessions are delivered through accessible platforms, taking into consideration internet connectivity limitations. Explore options for offline access or asynchronous participation to accommodate healthcare providers with limited internet access.
- *Tailor strategies based on local context and participants*: Tailor the training content to address the specific needs and context of the resource-constrained setting. Incorporate locally relevant case studies and practical examples to enhance applicability. Consider adapting the training curriculum to the local language or cultural context, if necessary.
- *Use mass media to increase uptake*: Promote the ECHO sessions through mass media channels and varied platforms to encourage broader reach, knowledge sharing, and collaboration. Foster a supportive environment where participants can learn from each other, exchange best practices, and build a community of practice.

## Discussion

We found that ECHO was a feasible and appropriate evidence-based intervention to address knowledge and capacity needs of CHEWs in relation to hypertension diagnosis and treatment. One of the identified critical facilitators was the presence and availability of experts and organizations with which to partner. KII participants emphasized that engagement with well-recognized experts not only enhances the program’s reputation, but also contributes to participants’ motivation and active engagement. Other research on Project ECHO has similarly identified the benefits of faculty recognition in promoting engagement with training sessions [10,21]. KII participants also underscored the level of effort involved in administering ECHO programs, which factored substantially into our decision to partner with mDoc Healthcare as opposed to facilitating the training program with our core Hypertension Treatment in Nigeria Program team.

Online training was viewed as both a barrier and facilitator compared to in-person sessions. While online platforms (such as Zoom) offer convenience and flexibility, there are challenges to overcome for sustained engagement. KII participants mentioned difficulties in obtaining case studies and encouraging active participation in live sessions. This suggests a need for consistent application of strategies to ensure meaningful engagement, such as incorporating interactive elements, utilizing multimedia resources, and promoting participant-driven discussions [21]. FGD participants consistently highlighted challenges with connectivity, which has been reported as a major barrier in similar settings [22,23].

The voluntary nature of participation emerged as both a benefit and a potential pitfall. While voluntary engagement allows for motivated and eager learners who actively seek knowledge, our discussions also highlighted challenges in consistent attendance and active engagement. Other ECHO programs and participants have similarly reported that time constraints and scheduling are primary barriers to participation [24,25,26,27]. Strategies such as incentives, recognition, and continuous communication may be useful in maintaining high participation rates and sustained engagement throughout the program.

HCWs in all sectors and locations face numerous competing demands, which are a barrier to long-term engagement. To encourage engagement, the hypertension ECHO program will seek national and international experts to facilitate didactic trainings, empanel the HCW participants in a WhatsApp group, and provide incentives for sustained participation.

## Limitations

A key limitation of this phase is that hypertension training may be offered by ECHO hubs that do not self-identify as cardiovascular or nephrology hubs. This may have biased our selection of hubs to survey and interview. Other ECHO hubs that were not included may offer trainings more suited to participants with lower levels of formal education akin to the target population for our hypertension ECHO series. Responses from ECHO hubs and willingness to participate may have varied based on location (primarily based in the United States), experience, or other factors which may limit application of these results within the Nigerian context and for implementation strategies for use with CHEWs. Despite these limitations, we were able to interview or contact 75% of existing ECHO hubs that focus on cardiovascular disease or nephrology, and have a set of rich interviews for analysis, including several hubs that offer trainings to community health workers. Further, our selection of implementation strategies and adaptation of the ECHO program was performed through a team-based process utilizing the expertise of our Nigerian collaborators. It is possible that some HCWs who participated in the FGDs may not have been willing to be open and honest in their responses. Selected primary healthcare centers and CHEWs may not have been representative of all HCWs participating in the Hypertension Treatment in Nigeria Program, and may therefore limit the generalizability of these results.

## Conclusion

The findings of this qualitative study provide valuable insights into the implementation of our proposed hypertension ECHO program for CHEWs in the Federal Capital Territory, Nigeria. These results were used to inform key decision points around: 1) scope and content of training, 2) format and frequency, 3) selection of implementation strategies, and 4) building a community of practice.

## Data Accessibility Statement

The datasets analyzed during the current study are not publicly available because the data collection, as approved by the Ethics Committee and Institutional Review Board, did not include having them become publicly available. The data can be made available to other researchers by contacting the corresponding author and with Ethics Committee and Institutional Review Board approval.

## Additional Files

The additional files for this article can be found as follows:

10.5334/gh.1277.s1 Online Supplement. Supplemental Figures 1–2 and Supplemental Tables 1–2.

10.5334/gh.1277.s2 Appendix. Appendix A to D.

10.5334/gh.1277.s3 COREQ Checklist. COREQ (COnsolidated criteria for REporting Qualitative research) Checklist.
