# Supplementary material for: Formative Evaluation and Adaptation of a Hypertension Extension for Community Health Outcomes Program for Healthcare Workers within the Federal Capital Territory, Nigeria
Source: Glob Heart. 2023 Nov 27;18(1):64. doi: 10.5334/gh.1277 (PMC10691284; doi:10.5334/gh.1277)
Supplement: Online Supplement. — Supplemental Figures 1–2 and Supplemental Tables 1–2. [file gh-18-1-1277-s1.pdf]

Supplemental Figure 1. Eligibility for Participation in the Hypertension ~~ECHO~~ Extension for Community Health Outcomes Program Based on Monthly Number of Participant Visits at Primary Healthcare Centers Participating in the Hypertension Treatment in Nigeria Program Between March 2020 to September 2021.

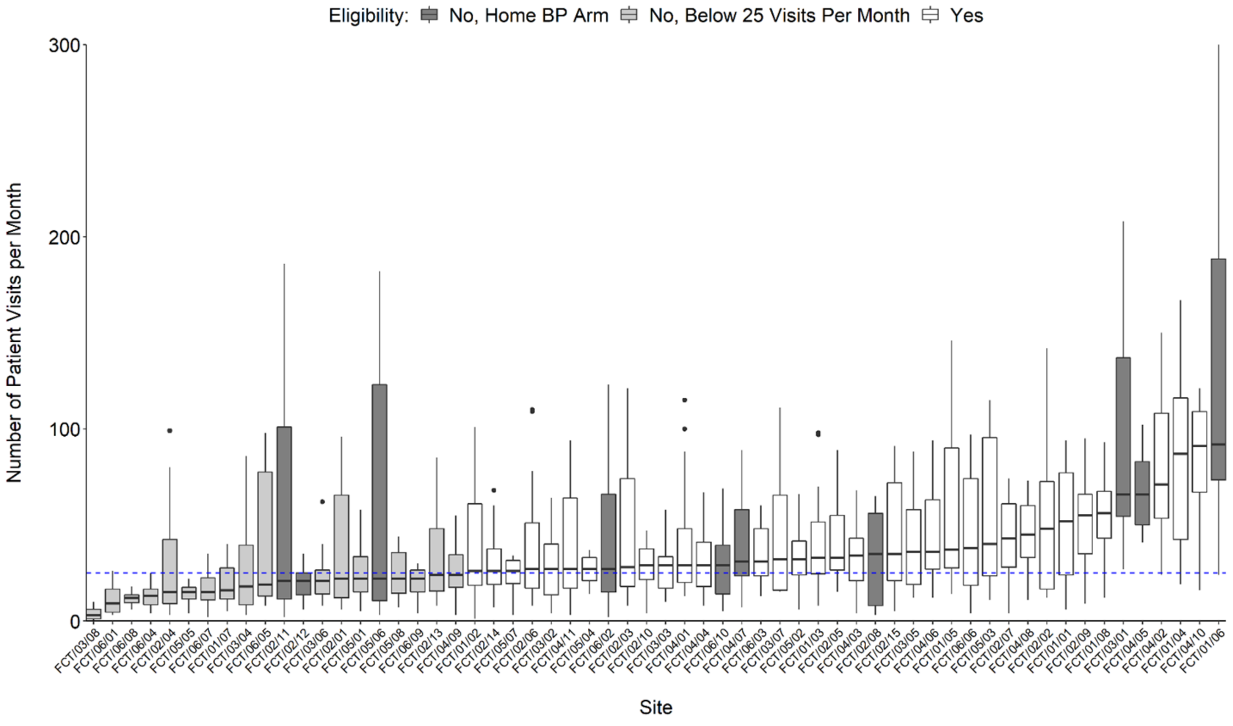

Abbreviations: BP, Blood Pressure

Supplemental Figure 2. Extension for Community Health Outcomes ~~ECHO~~ Hub Participants

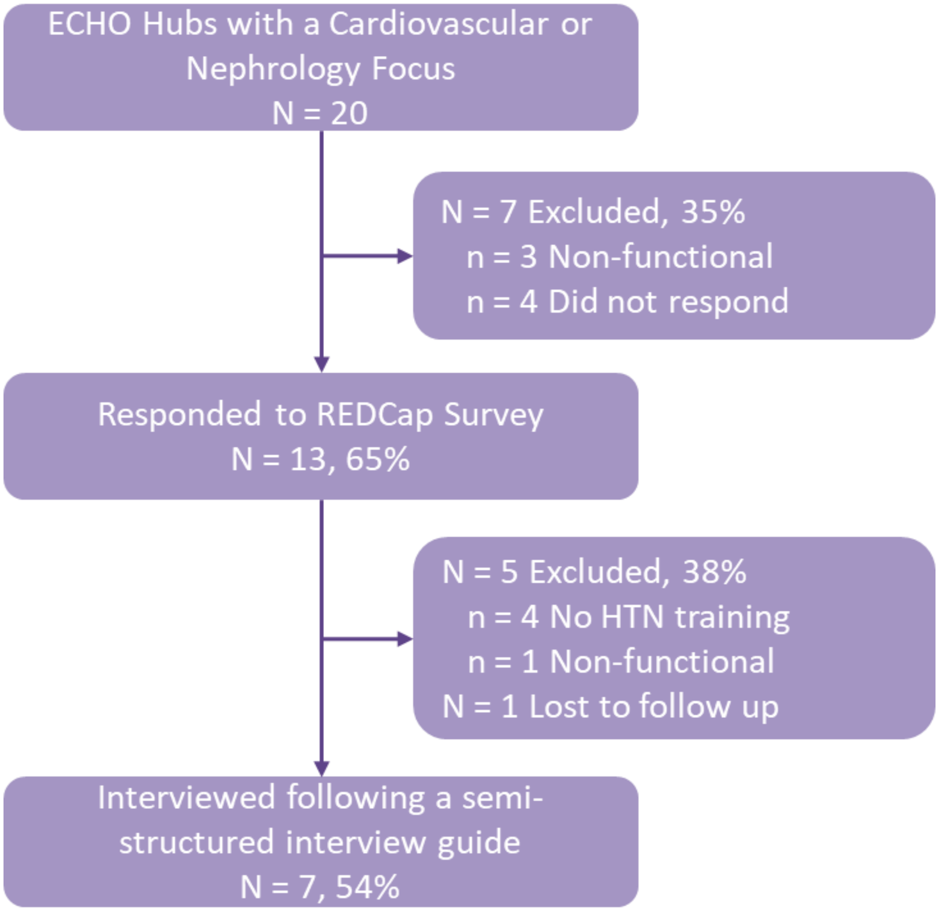

Abbreviations: ECHO, Extension for Community Health Outcomes; HTN, Hypertension; REDCap, Research Electronic Data Capture

Supplemental Table 1. Knowledge of Hypertension Diagnosis, Treatment, and Management Among Community Health Extension Workers in the Hypertension Treatment in Nigeria Program Before and After Initial Baseline Training.

	Pre-Test (n=73 CHEWs)		Post-Test[1] (n=63 CHEWs)	
**Question,**	**No. Responses**	**No. (% Correct)**	**No. Responses**	**No. (% Correct)**
Basic Questions				
Correctly identifying that all individuals with BP ≥ 140 and/or 90 mm Hg should be treated	72	55 (76.4)	62	54 (87.1)
Correctly identifying that hypertension treatment is necessary and affordable	71	45 (63.4)	63	45 (71.4)
Correctly identifying the ideal rest period for blood pressure measurement	73	70 (95.9)	62	61 (98.4)
Correctly identifying the ideal placement of the arm and cuff for blood pressure measurement	72	43 (59.7)	61	41 (67.2)
Successful monitoring of a clinic's effectiveness at treating patients is dependent on accurate blood pressure readings and treatment for hypertensive patients	70	61 (87.1)	62	49 (79.0)
Total Score Out of 5, mean (SD)		3.8 (1.1)		4.0 (1.1)
Advanced Questions				
Correctly identifying the appropriate treatment for patients with a history of heart attack within the previous three years	52	16 (30.8)	53	18 (34.0)
Correctly identifying the appropriate treatment for patients with a history of heart attack or stroke ever	53	19 (36.5)	53	14 (26.4)
Correctly identifying the appropriate treatment for patients who are or could become pregnant	49	18 (36.7)	49	13 (26.5)
Total Score Out of 3, mean (SD)		0.7 (1.0)		0.7 (1.0)

[1]Pre- and Post-tests were not linked by a participant identifier. Within participant changes in knowledge therefore cannot be measured.

Abbreviations: BP, Blood Pressure; CHEWs, Community Health Extension Workers; SD, Standard Deviation

Supplemental Table ~~3~~2. Characteristics of Frontline Healthcare Workers and Prior Extension for Community Health Outcomes Program Participants in Focus Group Discussions

Characteristic, No. (%)	No. Responses	Overall (n=42)	FGD1 (n=5)	FGD2 (n=5)	FGD3 (n=7)	FGD4 (n=6)	FGD5 (n=8)	FGD6 (n=5)	FGD7 (n=6)
Age, median (range)	42	42 (24-58)	42 (31-54)	47 (37-49)	35 (24-55)	39 (33-49)	39 (33-45)	44 (28-58)	49 (42-51)
Female	42	25 (59)	2 (40)	5 (100)	4 (57)	3 (50)	7 (88)	1 (20)	3 (50)
Role	42								
Community Health Extension Worker		30 (71)	5 (100)	3 (60)	7 (100)	3 (50)	7 (88)	5 (100)	0 (0)
Community Health Officer		3 (7)	0 (0)	2 (40)	0 (0)	0 (0)	1 (13)	0 (0)	0 (0)
Nurse		4 (10)	0 (0)	0 (0)	0 (0)	1 (17)	0 (0)	0 (0)	3 (50)
Record Officer		2 (5)	0 (0)	0 (0)	0 (0)	2 (33)	0 (0)	0 (0)	0 (0)
Medical Doctor		3 (7)	0 (0)	0 (0)	0 (0)	0 (0)	0 (0)	0 (0)	3 (50)
Highest Education Level	37								
High School Diploma		23 (62)	NR	1 (20)	7 (100)	3 (50)	8 (100)	4 (80)	0 (0)
Undergraduate Degree		10 (27)	NR	3 (60)	0 (0)	3 (50)	0 (0)	1 (20)	3 (50)
Post Graduate Degree		4 (11)	NR	1 (20)	0 (0)	0 (0)	0 (0)	0 (0)	3 (50)
Employed Full-Time	42	25 (59)	2 (40)	4 (80)	2 (29)	3 (50)	5 (63)	4 (80)	5 (83)
No. of people in household, median (range)	37	6 (1-20)	NR	6 (5-9)	5 (2-10)	5 (3-7)	7 (4-8)	6 (4-20)	5 (1-6)

Abbreviations: FGD, Focus Group Discussion; NR, Not Reported
