# Supplementary material for: Formative Evaluation and Adaptation of a Hypertension Extension for Community Health Outcomes Program for Healthcare Workers within the Federal Capital Territory, Nigeria
Source: Glob Heart. 2023 Nov 27;18(1):64. doi: 10.5334/gh.1277 (PMC10691284; doi:10.5334/gh.1277)
Supplement: Appendix. — Appendix A to D. [file gh-18-1-1277-s2.pdf]

# Review of TeleECHO Programs

Thank you for your willingness to complete this survey on behalf of your ECHO hub. We expect the survey will take 5-10 minutes to complete.

You are welcome to contact us at any time [abigailtice2020@u.northwestern.edu or abigail.baldridge@northwestern.edu] with any questions and may save your progress to return later as needed.

**Please complete the follow items:**

In what Country is this ECHO hub located?

__________________________________

In what City is this ECHO hub located?

__________________________________

What is the main source of funding for this hub?

Please choose one option.

- ◯ Government/Federal
- ◯ University
- ◯ Local non-governmental organization
- ◯ International non-governmental organization
- ◯ Other
- ◯ None

Please specify the Other source of funding:

__________________________________

Is there a secondary source of funding for this hub?

Please choose one option.

- ◯ Government/Federal
- ◯ University
- ◯ Local non-governmental organization
- ◯ International non-governmental organization
- ◯ Other
- ◯ None

Please specify the Other source of funding:

__________________________________

For how many years has this hub been functioning?

__________________________________

Is the hub currently offering training, educational opportunities, or otherwise interacting with learners in the community?

- ◯ Yes
- ◯ No, but plan to resume in the future
- ◯ No, with no plans to resume

Why is the ECHO hub not functioning at this time?

__________________________________

How many full-time staff work with the hub for purposes of organization and day-to-day function?

__________________________________

Please provide a range if you are uncertain.

How many part-time staff work with the hub for purposes of organization and day-to-day function?

__________________________________

Please provide a range if you are uncertain.

| | |
|---|---|
| How many volunteers (full or part-time) work with the hub for purposes of organization and day-to-day function?<br><br>Please provide a range if you are uncertain. | __________________________________ |
| Does your hub offer cardiovascular trainings? | ◯ Yes<br>◯ No |
| What types of cardiovascular trainings does your ECHO hub offer?<br><br>Please select all that apply. | ☐ Hypertension<br>☐ Heart Failure<br>☐ Imaging<br>☐ Coronary Artery Disease<br>☐ Rhythm Disorder<br>☐ Primary prevention<br>☐ Secondary prevention<br>☐ Other |
| Please specify the other cardiovascular trainings: | __________________________________ |
| Does your hub offer nephrology trainings? | ◯ Yes<br>◯ No |
| What types of nephrology trainings does your ECHO hub offer?<br><br>Please select all that apply. | ☐ Hypertension<br>☐ End Stage Renal Disease<br>☐ Chronic Kidney Disease<br>☐ Transplantation<br>☐ Hemodialysis<br>☐ Diabetes Mellitus<br>☐ Polycystic Kidney Disease<br>☐ Proteinuria<br>☐ Glomerular Disease<br>☐ Electrolyte and acid-base abnormalities<br>☐ Other |
| Please specify the other nephrology trainings: | __________________________________ |
| What mode(s) of communication (technology) do you utilize for trainings? | __________________________________ |
| Who participates in your trainings?<br><br>Please select all that apply. | ☐ Cardiologists<br>☐ Nephrologists<br>☐ Primary/ Family/Geriatric Care Physicians<br>☐ Emergency or Casualty Physicians<br>☐ Nurse Practitioners/Physician Assistants<br>☐ Nurses<br>☐ Technicians<br>☐ Community Health Workers<br>☐ Medical Trainees (i.e. students, residents, etc)<br>☐ Other |
| Please specify the other trainings participants: | __________________________________ |

# ECHO Hub: Interview Guide

**Welcome and Introduction:**
Hello, I am [INTRODUCTION].

My goal for this interview is to learn from you about your ECHO hub's hypertension trainings and operations. I am conducting interviews with other ECHO hubs, and intend to use this information to help inform implementation of an ECHO-like training program for community health extension workers in Nigeria who have recently begun managing hypertension for the first time. This study has been reviewed by the Northwestern University IRB and determined to be exempt as non-human subjects research.

We intend to summarize the information we learn in a publication for general knowledge, though we will not identify any respondents by name or location. We would like to audio record the interview so that we can make sure to accurately capture the thoughts, opinions, and ideas we hear from you. The records will be destroyed as soon as they are transcribed.

We do not believe there are any sensitive topics contained in this interview, but would like you to know that you may refuse to answer any question or stop the interview at any time.

Before we begin, are you willing for this interview to be recorded?

If yes: ☐ Start the recording

If no: ☐ Use this interview guide template to take notes

**Survey Review:**

If the participant completed a survey: Thank you for completing the electronic survey, I have your recorded responses.

If the survey is incomplete: Before we being the interview, can I ask you about the responses which were left blank?

☐ Attempt to complete these items if the participant is willing

If the participant did not complete the survey: I notice that you have not yet completed the survey. Did you have any trouble accessing it?

☐ Send the participant the survey link again, OR

☐ Fill it out together now

**Interview**

Staff expertise/Involvement:

1. Can you tell me about this Hub, including its major activities and partners?
    a. Can you describe what your role is within the ECHO hub?
    b. How long have you been involved with the ECHO hub?

2. Can you describe your background, including training and experiences that have prepared you for your role in this hub?
    a. *Probe: How has your training helped?*
    b. *Probe: How did your training lead you to this?*

3. Why was this ECHO hub created?

   a. *Probe: What were the gaps this ECHO hub was intended to fill?*
   b. Can you describe any barriers you encountered when initiating the hub? What about now?
   c. *Probe: How did you overcome those barriers?*

4. Can you describe how the hub was started?
   a. *Probe: Who was involved when this ECHO hub was started?*
   b. What skills or people were key for initial creation of the hub?
   c. What skills or people are critical for implementation of an ECHO hub which focuses on hypertension?

5. Can you describe the staff within your ECHO hub?
   a. Have your staff expanded? Who has been added?
   b. Are there any gaps in your staff or needs to keep the hub functioning well?
   c. How do changes in staff affect operation of the hub or dynamics?

<u>Hypertension Training/Immediate impact:</u>

6. Can you describe the contents of your hypertension training curricula?
   a. *Probe: Where did you get those? Did you create them from scratch or use existing resources?*
   b. What do you do to tailor your hypertension training curricula for your participants?
      i. *Probe: Who is responsible for changes to the curricula?*
      ii. *Probe: How do you adapt your trainings for people of different education levels?*
      iii. *Probe: How do you adapt your trainings for people with no formal training? Are you considering adaptions and curricula to tailor trainings for community health workers/workers without formal training?*

7. How did you initially identify knowledge gaps for hypertension?
   a. How do you assess knowledge/intervention gaps now to develop new trainings or revise your curricula?

8. How do you measure the effectiveness of your hypertension trainings?
   a. *Probe: Surveys, positive feedback, etc.*

9. How are your hypertension trainings delivered?
   a. *Probe: Are trainings delivered synchronously or asynchronously?*
   b. *Probe: Are your trainings provided individually or in a cohort style?*
   c. *Probe: do you find one works better than another?*

<u>Culture/Management:</u>

10. How does your hub function within the infrastructure of the university (or other affiliate)?
    a. *Probe: Is the hub governed/owned by the university?*
    b. *Probe: Who is your biggest supporter, how do you communicate needs and/or bring in new trainings? (getting to the point of how the hub is viewed within the organization—vital, ancillary, valued)*
    c. *Probe: How is the hub funded/sustained?*

11. How are participants held accountable to participate in the training?

12. Do you employ any motivators to keep people engaged in trainings?
    a. *Probe: Do you incentivize participants through things like compensation or certificates?*

13. If you were starting a new ECHO hub, or in my position, what is one recommendation you would have for me to start a hub? And to grow and sustain a hub?

That concludes the interview. Thank you very much for your time and responses. You are welcome to follow up with me at any time.

**FGD Guide: ECHO Participants**

**Introduction:**
Thank you for meeting with me today. My name is [Name]. I am working with the Cardiovascular Research Unit of University of Abuja and University of Abuja Teaching Hospital.

We are assessing the potential to implement a new training program for community health extension workers, which will link them with expert cardiologists and hypertension specialists and help CHEWs to care for hypertensive patients in their primary healthcare facilities. This educational program is delivered electronically through a platform like Zoom and includes both teaching and interaction by reviewing patient case studies presented by CHEWs. We plan to adapt the training program and conduct a 6-month pilot session with community health extension workers like you. The training program that we are interested in implementing is very similar to the ECHO program you recently participated in, though we plan to focus only on hypertension. We hope that the information we learn from this adaptation and pilot may be helpful to broader implementation of programs like the HTN Program across Nigeria.

This focus group discussion will take about 90 minutes. In order to get your valuable comments, I will be recording the session and taking some notes as well. Your responses will be kept confidential and we will ensure that any information to be included in our report does not identify you as the respondent. Do you have any questions before we begin?

**Focus Group Discussion:**

1. Please tell me about yourself and your background.

2. Please describe your experience in the ECHO program hosted by mDoc Healthcare.
    a. What challenges have you faced in participating?
    b. How has the training you have received through the ECHO program helped you?

<u>Intervention Characteristics</u>

**Relative Advantage**

3. How does the ECHO program compare to other similar existing educational programs in your primary health center?
    a. What advantages does the ECHO program have compared to existing programs?
    b. What disadvantages does the ECHO program have compared to existing programs?

**Complexity**

4. How complicated or difficult was the ECHO program for you?
    a. Is there anything that stood out to you in terms of duration, scope, and number of steps involved?
    b. Does the ECHO program reflect a clear departure from previous practices?

**Adaptability**

5. What kinds of changes or alterations do you think you will need to make to the ECHO program so it will work effectively for community health extensions workers in the HTN program?
    c. What kind of changes would be made to how the training program is delivered?
    d. What kind of changes would be made to the contents of the training program?

6. Are there components of the ECHO program that should not be altered?
    e. Which ones should not be altered?
    f. What are the reasons that they should not be altered?

**Cost**

7. Were there any costs incurred by you when you participated in the ECHO program?
    g. What were the costs?
    h. Were any of the costs too high? Which ones?

<u>Outer Setting</u>

**Patient Needs & Resources**

8. How well do you think the ECHO program will help CHEWs meet the needs of patients served by the HTN Program?
    i. In what ways will the ECHO program help CHEWs to meet their patients' needs? E.g., improved access to services? Reduced wait times? Help with self-management? Reduced travel time and expense?

**Cosmopolitanism**

9. To what extent do you network with other health workers, CHEWs, and physicians outside your primary health center?
    j. What are the venues?

10. Can you describe how participating in the ECHO Program helped you to network with others?
    k. Can you describe if the connections that you made were new or existing?

11. What kind of work- and hypertension-related discussions did you participate in through the ECHO Program that was helpful to you?
    l. Were there any things that were not helpful to you?

<u>Inner Setting</u>

**Tension for Change**

12. Do you see a strong need for this ECHO program among CHEWs who treat patients with hypertension in Nigeria PHCs?
    m. Why or why not?
    n. Do you believe others see a need for the ECHO program?

**Culture**

13. As a result of participating in the ECHO program, what ideas did you find you wanted to apply in your organization or for your patients?
    o. Can you describe an example?

**Compatibility**

14. How well did the ECHO Program fit with existing work processes and practices in your setting?
    p. What are likely challenges that may arise with an HTN ECHO Program?

**Organizational Incentives & Rewards**

15. What kinds of incentives do you think are needed to ensure that CHEWs participate in the ECHO program every month by calling in and actively engaging?

    q. What motivated you to participate in the ECHO program?

**Available Resources**

16. Did you have sufficient resources to participate in the ECHO program?
    r. [If yes] Are there any other resources that you received, or would have liked to receive?
    s. [If no] What resources were not available, and why?
    t. In terms of an HTN ECHO program, what resources are necessary?

**Leadership Engagement**

17. What kind of support or actions were necessary from leaders in your organization to help you successfully participate in the ECHO Program?
    u. What kind of support or actions from leaders in your organization were missing?

**Access to Knowledge & Information**

18. When you had questions about the ECHO program, who did you ask?
    v. How available were these individuals?

<u>Characteristics of Individuals</u>

**Knowledge & Beliefs about the Intervention**

19. Do you think the ECHO program will be effective in primary health centers engaged in the HTN Program?
    w. Why or why not?

**Self-efficacy**

20. How confident do you think your colleagues or other CHEWs in the HTN Program would feel about using the ECHO program?

<u>Process</u>

**Opinion Leaders**

21. Who are the key influential individuals to get on board with development and roll out of an ECHO program for hypertension education among CHEWs?

**FGD Guide: Community Health Extension Workers**

**Introduction:**
Thank you for meeting with me today. My name is [NAME]. I am working with the Cardiovascular Research Unit of University of Abuja and University of Abuja Teaching Hospital.

We are determining how to adapt and implement a new hypertension training program for community health extension workers in Abuja, Nigeria. The training program will link CHEWs in public primary health centers with expert cardiologists and hypertension specialists. This educational program is delivered electronically through a platform like Zoom and includes teaching by the cardiologists and specialists and review of patient case studies presented by CHEWs. CHEWs who participate in the training program may join a training session, on a platform like Zoom, once a month. A training session might include hearing from an expert how to manage hypertension during pregnancy, and then review of the diagnosis and treatment of an actual patient. We plan to adapt the training program and conduct a 6-month pilot session with community health extension workers like you. We hope that the information we learn from this adaptation and pilot may be helpful to broader implementation of programs like the HTN Program across Nigeria.

This focus group discussion will take about 90 minutes. In order to get your valuable comments, I will be recording the session and taking some notes as well. Your responses will be kept confidential and we will ensure that any information to be included in our report does not identify you as the respondent. Do you have any questions before we begin?

**Focus Group Discussion:**

1. Please tell me about yourself and your experience as a community health extension worker.

2. Please describe how you have been involved in the Hypertension Treatment in Nigeria Program.
    a. What challenges have you faced in detecting, treating, or managing patients with high blood pressure?

<u>Intervention Characteristics</u>
**Adaptability**

3. What hypertension trainings have you participated in before, or that you are participating in now?
    a. How has the training you have received through the HTN Program helped you?
    b. What was helpful about the hypertension training programs you participated in before?
    c. What could be improved about this training for other CHEWs?

4. Are there components of hypertension training programs that should not be altered?
    a. Which ones should not be altered?
    b. What are the reasons that they should not be altered?

5. Please describe the hypertension training you need in order for you and your primary health center to treat hypertensive patients moving forward.
    a. How do you think training should or could be delivered?
    b. What should the contents of the training be?
    c. What more do you wish you knew?

Outer Setting

**Patient Needs & Resources**

6. How well do you think the hypertension training you have received helps you to meet the needs of patients served by your primary health center?
    a. In what ways has the hypertension training you have received helped you to meet your patients' needs? E.g., improved access to services? Reduced wait times? Help with self-management? Reduced travel time and expense?
    b. How could the hypertension training be improved to help you better meet your patients' needs?

**Cosmopolitanism**

7. To what extent do you network with other CHEWs and physicians outside your primary health center?
    a. What are the venues?

8. What kind of work- and hypertension-related discussions do you have with others outside your setting?
    a. What professional networking do you engage in? Local or national conferences? Trainings?

Inner Setting

**Networks & Communications**

9. When you have a question about how best to treat or manage a patient with hypertension, who are your "go-to" people?
    a. Can you describe a recent example when you sought help within your PHC?
    b. Can you describe a recent example when you sought help outside of your PHC?

**Tension for Change**

10. Do you see a strong need for ongoing hypertension training among CHEWs who treat patients with hypertension in Nigeria PHCs?
    a. Why or why not?
    b. Do you believe others see a need for ongoing hypertension training?

**Organizational Incentives & Rewards**

11. What kinds of incentives do you think are needed to ensure that CHEWs participate in ongoing hypertension training?
    a. What incentives would be needed if CHEWs were asked to participate in monthly online training?

**Learning Climate**

12. To what extent do you feel like you can try new things to improve how you care for hypertensive patients?
    a. Do you feel like you have the time and energy to think about ways to improve how you care for patients with hypertension?

**Available Resources**

13. What resources would you need in order to participate in additional hypertension training?

Characteristics of Individuals

**Knowledge & Beliefs about the Intervention**

14. Do you think a monthly online hypertension training program will be effective in your primary health center?
    a. Why or why not?

15. How do you feel about the monthly online hypertension training program being used in your primary health center?
    a. Do you have any feelings of anticipation? Stress? Enthusiasm? Why?

**Self-efficacy**

16. How confident are you that you will be able to participate in a monthly online hypertension training program?
    a. What gives you that level of confidence (or lack of confidence)?
    b. What challenges do you foresee that make you feel less confident about participating?

17. How confident do you think your colleagues would feel about participating in a monthly online hypertension training program?
    a. What gives them that level of confidence (or lack of confidence)?
    b. What challenges do you foresee that make you feel less confident about your colleagues participating?
